# Human Serum-Specific Activation of Alternative Sigma Factors, the Stress Responders in *Aggregatibacter actinomycetemcomitans*

**DOI:** 10.1371/journal.pone.0160018

**Published:** 2016-08-04

**Authors:** Gaoyan Tang-Siegel, Roger Bumgarner, Teresa Ruiz, Weerayuth Kittichotirat, Weizhen Chen, Casey Chen

**Affiliations:** 1 Department of Molecular Physiology and Biophysics, College of Medicine, University of Vermont, Burlington, VT, United States of America; 2 Department of Microbiology, University of Washington, Seattle, WA, United States of America; 3 Systems Biology and Bioinformatics Research Group, Pilot Plant Development and Training Institute, King Mongkut's University of Technology Thonburi, Bangkok, Thailand; 4 Ostrow School of Dentistry, University of Southern California, Los Angeles, CA, United States of America; Bose Institute, INDIA

## Abstract

*Aggregatibacter actinomycetemcomitans*, a known pathogen causing periodontal disease and infective endocarditis, is a survivor in the periodontal pocket and blood stream; both environments contain serum as a nutrient source. To screen for unknown virulence factors associated with this microorganism, *A*. *actinomycetemcomitans* was grown in serum-based media to simulate its *in vivo* environment. Different strains of *A*. *actinomycetemcomitans* showed distinct growth phenotypes only in the presence of human serum, and they were grouped into high- and low-responder groups. High-responders comprised mainly serotype c strains, and showed an unusual growth phenomenon, featuring a second, rapid increase in turbidity after 9-h incubation that reached a final optical density 2- to 7-fold higher than low-responders. Upon further investigation, the second increase in turbidity was not caused by cell multiplication, but by cell death. Whole transcriptomic analysis via RNA-seq identified 35 genes that were up-regulated by human serum, but not horse serum, in high-responders but not in low-responders, including prominently an alternative sigma factor *rpoE* (**σ**^E^). A *lacZ* reporter construct driven by the 132-bp *rpoE* promoter sequence of *A*. *actinomycetemcomitans* responded dramatically to human serum within 90 min of incubation only when the construct was carried by a high responder strain. The *rpoE* promoter is 100% identical among high- and low-responder strains. Proteomic investigation showed potential interactions between human serum protein, e.g. apolipoprotein A1 (ApoA1) and *A*. *actinomycetemcomitans*. The data clearly indicated a different activation process for *rpoE* in high- *versus* low-responder strains. This differential human serum-specific activation of *rpoE*, a putative extra-cytoplasmic stress responder and global regulator, suggests distinct *in vivo* adaptations among different strains of *A*. *actinomycetemcomitans*.

## Introduction

The gram-negative, non-motile, capnophilic bacterium *Aggregatibacter actinomycetemcomitans* is a recognized oropharyngeal colonizer, found in the oral cavity of 20% of the population [1]. However, individuals who are colonized with *A*. *actinomycetemcomitans* range from being healthy to developing into aggressive periodontitis [2] and/or systemic infections, including infective endocarditis [3] and pulmonary infections [4].

Different consequences of *A*. *actinomycetemcomitans* colonization could be attributed to different individuals’ immune responses, and/or to heterogeneity of *A*. *actinomycetemcomitans*. Up to now, seven different serotypes of *A*. *actinomycetemcomitans* strains, serotypes a to g, have been identified according to their distinct O-polysaccharide (O-PS) structures of lipopolysaccharides [5–9]. However, heterogeneity among different *A*. *actinomycetemcomitans* strains goes beyond the differences in the O-PS gene clusters [10–12]. For example, different forms of collagen adhesin EmaA (Extracellular matrix adhesin A), a virulence factor of *A*. *actinomycetemcomitans* were identified in different serotypes [10,13]. Our earlier work though comparative genomic analysis identified 3,301 genes in the pangenome of *A*. *actinomycetemcomitans*, including 2034 “core genes” that are present in all strains and 1,267 “accessory genes” that are not present in all strains of this species [11]. Whole genome sequencing and phylogenetic analysis identified five clades associated with *A*. *actinomycetemcomitans*: Clade b (serotype b), Clade c (serotype c), Clade e/f (serotype e and f), Clade a/d (serotype a and d), and Clade e' (serotype e strains) [12]; and the differences between strains were between 0 and 16.7% [12]. [11]In addition, certain strain of *A*. *actinomycetemcomitans*, e.g. D11S-1, carries large plasmids and is infected by bacteriophages [14]. Such extra genetic information carried by plasmids and bacteriophages may also change bacterial growth fitness and virulence [15, 16]. Therefore, we hypothesize that accessory genes and mobile genetic elements including plasmids and phages modulate the fitness and virulence of *A*. *actinomycetemcomitans in vivo*, eventually leading to disparate consequences of infection by this organism in human hosts.

There are at least two important *in vivo* growth environments to be considered in the investigation of *A*. *actinomycetemcomitans* pathogenesis: periodontal pockets and the blood stream. The periodontal pockets are filled with inflammatory exudate from serum [17]. In addition, *A*. *actinomycetemcomitans* causes extra-oral infections [3] indicating that this microorganism is able to survive and mobilize from oral sites to extra-oral sites in the blood stream. Therefore, human serum was chosen as the base of growth media.

In this study, high- and low-responder groups were initially identified based on strains’ responses to human serum. The high-responder strains, largely limited to serotype c, exhibited a “diauxic-like” growth phenomenon in the presence of human serum, featuring an initial logarithmic rise in turbidity starting at 3–4 hour; and a second rapid increase after 9-hour exposure to human serum. However, the second increase of turbidity was found associated with cell death. We further investigated gene expression and protein expression at the transcriptional and translational levels respectively, of high- and low-responders to human serum. The transcriptomics, proteomics and genetic data demonstrated that human serum, but not horse serum, activated an alternative sigma factor *rpoE* (**σ**^E^ or **σ**^24^) only in the high-responder strain. The data suggest that the activation of *rpoE in vivo* is different in the high- *versus* low-responder strains of *A*. *actinomycetemcomitans*.

## Materials and Methods

### Bacterial strains and plasmids

Twenty-five strains of *A*. *actinomycetemcomitans* representing serotypes a-f were chosen for this study (Table 1). The majority were clinical strains isolated from the periodontal pockets of patients with periodontitis. The RhAA1 strain was isolated from a rhesus macaque, an Old World primate [18]. *A*. *actinomycetemcomitans* strains were recovered on TSBYE agar containing 3% trypticase soy broth, 0.6% yeast extract and 1.5% agar (Becton Dickinson and Company) and incubated statically in a 37°C incubator with 5% humidified carbon dioxide. All plasmids were purified from *Escherichia coli* grown in broths containing 1% BactoTryptone, 0.5% yeast extract and 1% sodium chloride (Lysogenic broth, LB) with appropriate antibiotics at 37°C under aerobic conditions with agitation.

**Table 1:** A list of 25 strains of *A*. *actinomycetemcomitans*, their serotypes and responses to human serum.

Phenotypes/Serotypes	Strains
High-responders	
c	D11S-1, D17P-2, 2302, D15P-1, D37P-1, D45P-1, 2444, AAS12
f	D18P-1
Low-responders	
a	D7S-1, D17P-3, ATCC29523*, 283, SA286, SA558
b	ANH9381, RhAA1**, ATCC29524*, Y4*, SCC1398
c	CCUG35886 ***, ATCC33384*
d	163B
e	SCC393, SC1083

*ATCC strains and Y4 were purchased from American Type Culture Collection.

** RhAA1 was isolated from a rhesus macaque, an Old World primate.

*** CCUG35886, a strain from Culture Collection of the University of Göteborg, Sweden, was originally isolated from the blood sample of a patient with infective endocarditis.

### Growth phenotype analysis using serum-based media and Bio-Screen C automated microbiology growth curve analysis system

*A*. *actinomycetemcomitans* strains were recovered from a -80°C freezer and grown on TSBYE agar plates for 48–72 h. One colony of each strain was transferred to a polystyrene culture tube containing 6 ml TSBYE broth, grown statically for 20–23 h, and re-suspended in fresh serum-based growth medium with a starting cell number equivalent to 0.5 × 10^8^–1.0 × 10^8^ cells/ml. Serum-based media were prepared by mixing TSBYE with 50% serum from either humans (Cat # H3667), bovines (Cat # F4135), swine (Cat # P9783) or sheep (Cat # S2263), all obtained from Sigma-Aldrich (St Louis, MO); or horse (Cat # 262–500), obtained from QUAD FIVE (Ryegate, MT). The human serum was pooled male type AB plasma. All sera used in this study were heat-inactivated at 56°C for 30 min. The serum was filtered with a 0.22 μm filter before use to remove large protein precipitates. The prepared aliquots of 300 μl of bacterial suspension in fresh serum-based media were loaded into each well of a 100-well honeycomb microplate, and covered with 20 μl of sterile mineral oil to create an anaerobic environment. Turbidity of the broth culture in each well was measured continuously using a wide band filter of 420–580 nm, with continuous shaking, and 48-h growth curves were obtained using a Bio-Screen C automated microbiology growth curve analysis system (Growth Curve USA, Piscataway, NJ).

### Growth phenotype analysis based on extracted total DNA and colony forming units

Three strains, D7S-1 [19], SCC1398 [11] and D11S-1 [14], representing serotypes a, b, and c, were chosen as representative strains (Table 2). The 23-h growth curves of *A*. *actinomycetemcomitans* in three different broths (TSBYE, TSBYE + 50% horse serum, or TSBYE + 50% human serum) were established based on their static growth in a 37°C incubator with 5% humidified carbon dioxide atmosphere. The total extracted DNA and colony forming units (CFUs) were determined at 3-h to 4-h intervals. Total DNA was extracted using the QIAamp DNA Mini Kit (Qiagen, Valencia, CA). Briefly, bacteria in culture (≤ 2 × 10^9^ cells) were collected by centrifugation in a microcentrifuge, re-suspended in lysis buffer ATL, and incubated with proteinase K overnight, followed by incubation with RNase A at room temperature for 2 min. The bacterial lysate was cleaned by passage through spin columns, and purified DNA was eluted in AE buffer (10 mM Tris Cl, 0.5 mM EDTA). DNA concentration was measured using a NanoPhotometer (P360, Implen GmbH, Germany). Recovered CFUs were determined by applying serial dilutions to fresh TSBYE agar plates. The data were analyzed using un-paired *t* test with GraphPad Prism 6 software, *P* < 0.05 were considered statistically significant.

**Table 2:** Representative strains and plasmids.

Strains	Genotype/Remarks	Sources and references
*A*. *actinomycetemcomitans*		
D11S-1	A clinical serotype c strains, isolated from the diseased site of a 16-year-old African American with generalized aggressive periodontitis	[14]
D11S-1S	A non-fimbriated variant strain of D11S-1	This study
D11S-1S/pJT5/*rpoE*P	D11S-1S transformed with plasmid pJT5/*rpoE*P	This study
D11S-1S/PNP3	D11S-1S transformed with plasmid PNP3	This study
SCC1398	A clinical serotype b strain, isolated from the diseased site of a 25-year-old Caucasian with localized aggressive periodontitis	[11]
SCC1398S	A non-fimbriated variant of SCC1398	This study
SCC1398S/pJT5/*rpoE*P	SCC1398S transformed with plasmid pJT5/*rpoE*P	This study
D7S-1	A clinical serotype a strain, isolated from the diseased site of a 29-year-old African American with generalized aggressive periodontitis	[19]
D7S-1S	A non-fimbriated variant of D7S-1	This study
*E*. *coli*		
*ccd*B Survival™ 2 T1R	F-*mcr*A Δ(*mrr*-*hsd*RMS-*mcr*BC) Φ80*lac*ZΔM15 Δ*lac*X74 *rec*A1 *ara*Δ139 Δ(*ara*-*leu*)7697 *gal*U *gal*K *rps*L (StrR) *end*A1 *nup*G *fhu*A::*IS2*	Invitrogen, Carlsbad, CA
Plasmids		
pJT5	A promoterless *lacZ* reporter construct, Kan^R^	[20]
pJT5/*rpoE*P	The 132-bp putative promoter sequence of *rpoE* was ligated into pJT5, Kan^R^	This study
pNP3	Plasmid pMMB67 carrying the green fluorescent protein (GFP), controlled by two promoters: the promoters of spectinomycin and *tac*. Amp^R^	[21]

### Bacterial preparation for total RNA extraction

Bacterial colonies were transferred from TSBYE agar plates to polystyrene culture tubes containing 6 ml of either TSBYE, TSBYE + 50% horse serum, or TSBYE + 50% human serum. After 22 h of growth, *A*. *actinomycetemcomitans* attached tightly to the tubes, and the supernatant was decanted and replaced with fresh TSBYE, TSBYE + 75% horse serum, or TSBYE + 75% human serum, respectively, to maintain the same serum exposure conditions. Bacteria were allowed to grow for an additional 6 h, then collected using cell scrappers, washed with phosphate buffered saline pH 7.4 (PBS, 10 mM sodium phosphate, 150 mM sodium chloride), and pelleted at 8,000×g at 4°C for 10 min. Total RNA was extracted using the Ribo-Pure Bacterial RNA isolation kit, based on the manufacturer’s instructions (Life Technology, Grand island, NY). Briefly, 1.0×10^9^ cells were lysed using zirconia beads, and the lysate was mixed with chloroform. The RNA was extracted in the top aqueous phase, cleaned and treated with DNase to prepare for RNA sequencing.

### Transcriptomic analysis

The purified mRNA was fragmented using divalent cations at elevated temperature. Cleaved RNA fragments were copied into first strand cDNA using reverse transcriptase and random primers, followed by second strand cDNA synthesis using DNA polymerase I and RNase H. cDNA products were purified and enriched by PCR to create a final cDNA library, according to TruSeq stranded total RNA sample preparation kit (Illumnia, San Diego, CA). After sequencing, the reads for each sample were mapped to the corresponding genomes for each strain using the Geneious software (Biomatters LTD, Auckland, New Zealand). After mapping, the average coverage (number of sequences/nucleotide) was calculated for each predicted gene. Coverage was normalized by averaging across all genes for each sample and scaled up by multiplying by a factor of 1000. All transcriptomic data were generated based on duplicate experiments. The transcriptomic analysis was performed in a core facility, located in the University of Washington, Seattle, WA.

### Cloning using the putative promoter sequence of *rpoE* to drive the expression of *lacZ*

The alternative sigma factor *rpoE* (σ^24^), together with *rseA*, *rseB* and *rseC* is located within a putative four-gene operon (*rpeE-rseABC*) in *A*. *actinomycetemcomitans*. The putative 132 bp promoter sequence of this operon was amplified from the D11S-1 strain using a high-fidelity polymerase (Invitrogen, Carlsbad, CA), and engineered with restriction sites for *KpnI* and *BamHI* (New England Biolabs, Ipswich, MA), using primers *KpnI*-*rpoE*P (5’-CC***GGTACC***TTTATAATGAATTAGTTCTC-3’) and *rpoE*P-*BamHI* (5’- ***GGATCC***ATAACCTCATAATCACGTCTA-3’). The 146 bp amplicon was cloned into a pCR4 TOPO Vector (Invitrogen, Carlsbad, CA), digested with *KpnI* and *BamHI*, and re-ligated into a promoter-less *lacZ* reporter construct pJT5 [20]. The new constructed plasmid pJT5/*rpoE*P was transformed into two *A*. *actinomycetemcomitans* strains D11S-1 and SCC1398 by electroporation to generate two new strains: D11S-1S/pJT5/*rpoE*P and SCC1398S/pJT5/*rpoE*P [22]. After electroporation, the bacteria were recovered by plating on TSBYE plates, supplemented with 50 μg/ml Kanamycin and 40 μg/ml X-gal (5-bromo-4-chloro-3-indolyl-β-D-galactopyranoside).

### β-Galactosidase assay

β-galactosidase, catalyzes the hydrolysis of ortho-nitrophenyl-β-D-galactopyranosidase (ONPG), converting ONPG into lactose and ortho-nitrophenol (ONP) [23]. This assay was used to determine quantitatively the response of the 132 bp *proE* promoter of *A*. *actinomycetemcomitans* to either human serum or hyperosmotic stress in *E*. *coli*. Hyperosmotic stress was generated by supplement of sucrose to the LB medium to yield a final concentration of 0.464 M [24]. The assay was modified based on the instructions of the β-Gal assay kit (Invitrogen, Carlsbad, CA). *E*. *coli* harboring the plasmid pJT5/*proE*P were grown overnight in LB with 50 μg/ml kanamycin, and diluted to approximate 1.0 × 10^8^ cells/ml in LB, LB + 0.464 M sucrose, or LB + 50% human serum. Cell aliquots were collected at 30 min intervals. Bacteria were washed with PBS pH 7.4, and re-suspended in 0.25 M Tris lysis buffer (pH 8.0). Cells were lysed by 10 repetitions of alternatively freezing in a dry ice/ethanol bath and thawing in a 37°C water bath, with vigorous vortexing between the freezing and thawing cycles. The final quantification was based on the following equations:

Activity ofβ-galactosidase=nmoles ONPG hydrolyzed/t/mg protein

$$\text{nmoles of ONPG hydrolyzed}=\left({\text{OD}}_{420}\right)(8\times 10^{5}\;\text{nanoliters})\;/\;(4500\;\text{nl/nmoles}\times \text{cm})\left(1\text{cm}\right)$$

In these equations, 4500 is the extinction coefficient, t = the time of incubation at 37°C, and mg protein = the amount of protein lysate used for the assay, determined based on A_280_ readings.

*A*. *actinomycetemcomitans* strains D11S-1S, D11S-1S/pJT5/*rpoE*P, SCC1398S, and SCC1398/pJT5/*rpoE*P were grown overnight in TSBYE with/without 50 μg/ml kanamycin. The bacteria were then diluted to 0.5 × 10^8^–1.0 × 10^8^ cells/ ml and grown in 50% human serum, with samples collected at 90 min intervals, and prepared for the β-galactosidase assay.

### Transmission electron microscopy (TEM)

The fimbriated strain D11S-1 was recovered from a -80°C freezer, grown on a TSBYE plate for 3 d, sub-cultured on three different fresh agar plates (TSBYE, TSBYE + 50% horse serum, or TSBYE + 50% human serum), and grown for additional 60 h. Cells were recovered from the plates with minimal perturbation. Briefly, one droplet of 5 μl PBS buffer was deposited over a group of colonies and a second droplet of 3 μl buffer was deposited on a 400 mesh carbon-coated grid previously rendered hydrophilic. Two droplets were allowed to make contact for 20 sec, after which 3 μl of buffer was added to the grid to prevent drying of the transferred colonies. To remove small debris while exerting minimal stress on the bacteria, the grids were rinsed twice with 6 μl of buffer, wicking off the excess liquid with #40 Whatman paper in between washes. Grids were subsequently stained by first wicking off the excess liquid as before and immediately adding 6 μl of Nano-W staining solution (Methylamine Tungstate, Nanoprobes, Yaphank, NY). This step was repeated 3 times, not allowing the grid surface to dry at any time in the process and the last drop of stain was left on the grids for 30–60 sec. Finally, the stain was wicked off completely and the grids were slowly air-dried. Data was collected using a Tecnai12 electron microscope (FEI, Hillsboro, OR) equipped with a LaB6 cathode (Kimball Physics, Wilton, NH), a 14 μm, 2048 by 2048 pixel CCD camera (TVIPS, Gauting, Germany) and a dual-axis tomography holder (Fischione, Export, PA). 0° images were recorded at an acceleration voltage of 100 kV at nominal magnifications ranging from × 2,700 to × 42,000, which correspond, respectively, to 4.66 nm and 0.31 nm pixel sizes on the specimen scale.

### Confocal laser scanning microscopy

The non-fimbriated strain D11S-1S was transformed by electroporation with the plasmid pNP3 that expresses green fluorescent protein (GFP) [25]. The strain was recovered from -80°C, grown on a TSBYE agar plate with 50 μg/ml ampicillin for 48 h, sub-cultured in three different broths (TSBYE, TSBYE + 50% horse serum, or TSBYE + 50% human serum) with 50 μg/ml ampicillin, and grown for additional 23 h. Twenty microliter of bacterial suspension from each sub-culture was spread directly on a glass slide, air-dried slowly at room temperature, and observed using a confocal laser scanning biological microscope (FluoView FV10i, Olympus, Center Valley, PA, USA).

### Sodium dodecyl sulfate-polyacrylamide gel electrophoresis (SDS-PAGE)

Bacteria were grown in polystyrene culture tubes containing 6 ml of TSBYE, 50% horse serum in TSBYE, or 50% human serum in TSBYE respectively. After 23-h incubation, the supernatant was decanted; the attached bacteria were washed twice with 6 ml of sterile PBS and re-suspended in 10 mM 4-(2-Hydroxyethyl) piperazine-1-ethanesulfonic acid buffer (HEPES, pH 7.4). Approximately equal numbers of bacteria from each sample were mixed with the loading buffer containing: 2% SDS, 5% (v/v) β-mercaptoethanol, 10% (v/v) glycerol, 0.02% (w/v) bromophenol blue and 0.06 M Tris pH 6.8; boiled for 5 min [26]; and loaded in a 4–15% gradient polyacrylamide Mini-PROTEAN TGX precast gel (Bio-Rad, Hercules, CA). Electrophoresis was run in Laemmli buffer [27] at 80 V, 4°C. The gel was fixed with 50% methanol, 10% acetic acid for 10 min, incubated in colloidal blue stain (Invitrogen, Carlsbad, CA) with agitation for 3 h, and de-stained in deionized water for 8 h. Bands of interest were excised for liquid chromatography/mass spectrometry analysis.

### Liquid chromatography/mass spectrometry (LC/MS) analysis

Excised gel bands were subjected to in-gel trypsin digestion, dehydration with acetonitrile, and rehydration with 50 mM ammonium bicarbonate. Digested peptides were extracted by removing the ammonium bicarbonate solution [28]. Samples were analyzed using an LC/MS system consisting of an Eksigent NanoLC Ultra 2D (Dublin, CA) and Thermo Fisher Scientific LTQ Orbitrap XL (San Jose, CA). Proteome Discoverer 1.4 (Thermo Fisher Scientific, San Jose, CA) and Sequest algorithms were used for protein identification. Only peptides with a minimum length of six amino acids were considered for identification. Identifications of peptides were also validated by manual inspection of the mass spectra. The LC/MS analysis was performed in the proteomics core facility located at the University of Southern California, Los Angeles, CA.

## Results

### Distinct growth phenotypes of *A*. *actinomycetemcomitans* were only identified in the presence of human serum

**Fig 1:**
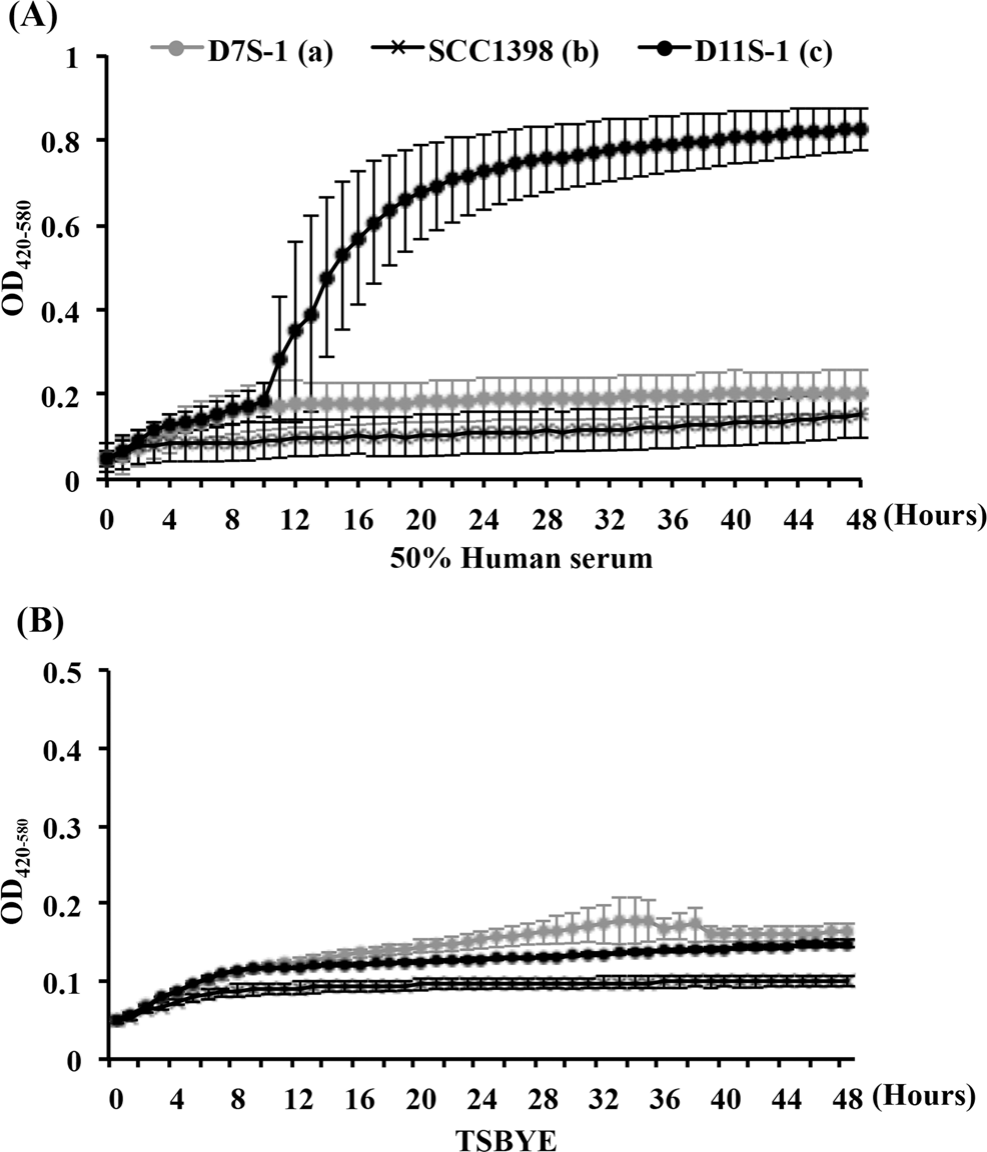
Comparison of growth curves of three fimbriated strains: D11S-1, D7S-1, and SCC1398, based on optical density (OD), corresponding to turbidity changes. **(A).** Continuous 48-h OD readings of three strains grown in trypticase soy broth with yeast extract (TSBYE) + 50% human serum. The high-responder strain D11S-1 shows a growth phenomenon, featuring an initial logarithmic phase starting 3–4 h, and a second rapid increase of OD readings after 9-h exposure to human serum, reaching a final OD 3- to 7-fold higher than two low-responder strains. **(B).** Continuous 48-h OD readings of three strains grown in TSBYE. Very similar growth curves for all three strains were observed under this condition.

Twenty-five strains, representing serotypes a to f were chosen to investigate growth fitness in a condition closely resembling the *in vivo* growth environment in humans (Table 1). This condition was provided using TSBYE mixed with 50% human serum as the base of growth medium. Two major phenotypes were identified by monitoring changes in turbidity of the culture medium, termed high- and low-responders according to their distinct responses to human serum. The high-responder strains showed a growth phenomenon, characterized by an initial logarithmic phase starting at 3–4 h, followed by a second rapid increase of turbidity 9 h after exposure to human serum, which reached a final optical density (OD) 2- to 7-fold higher than the low-responder strains grown under the same conditions (Fig 1A). The second rapid increase in OD was not observed when the same strains were grown in TSBYE alone (Fig 1B) or in the presence of horse serum. Among 25 strains, nine were high-responders including eight serotype c strains and one serotype f strain; 16 low-responders included six serotype a, five serotype b, two serotype c, one serotype d, and two serotype e strains (Table 1). One low-responder, CCUG35886 (serotype c) was originally isolated from the blood sample of a patient with infective endocarditis (Table 1).

**Fig 2:**
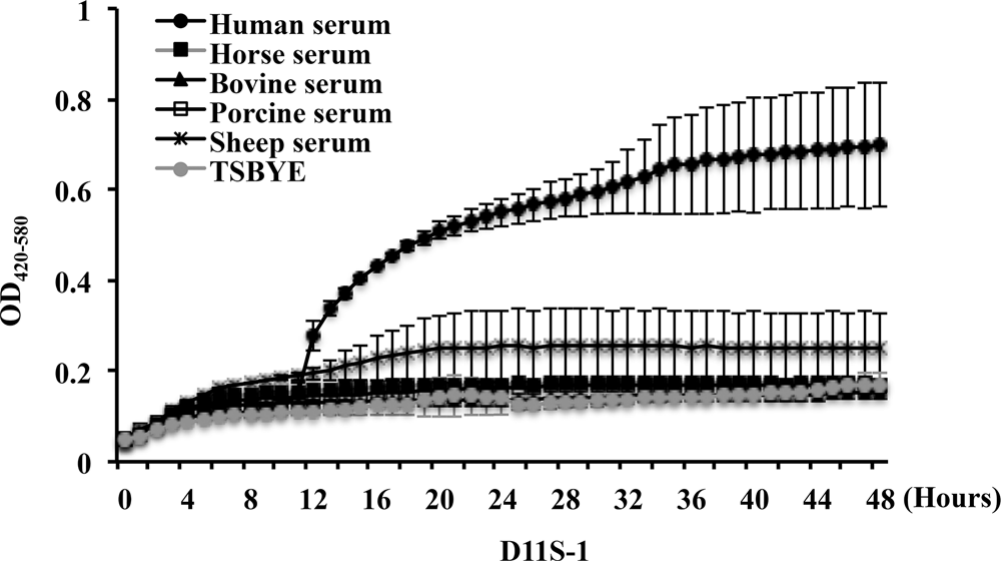
Comparison of growth curves of strain D11S-1 in sera from different animals. Continuous 48 h OD readings of D11S-1 grown in TSBYE + 50% of serum from humans, horses, bovines, swine, or sheep show that the unusual growth phenomenon was only observed in the presence of human serum.

Further investigations using sera from other animal sources demonstrated that this unusual growth phenomenon was only stimulated by human serum, but not by horse, bovine, porcine or sheep serum, indicating that this is a human serum-specific phenomenon (Fig 2).

### The second rapid increase of turbidity observed for the high-responder strain was caused by cell deterioration

**Fig 3:**
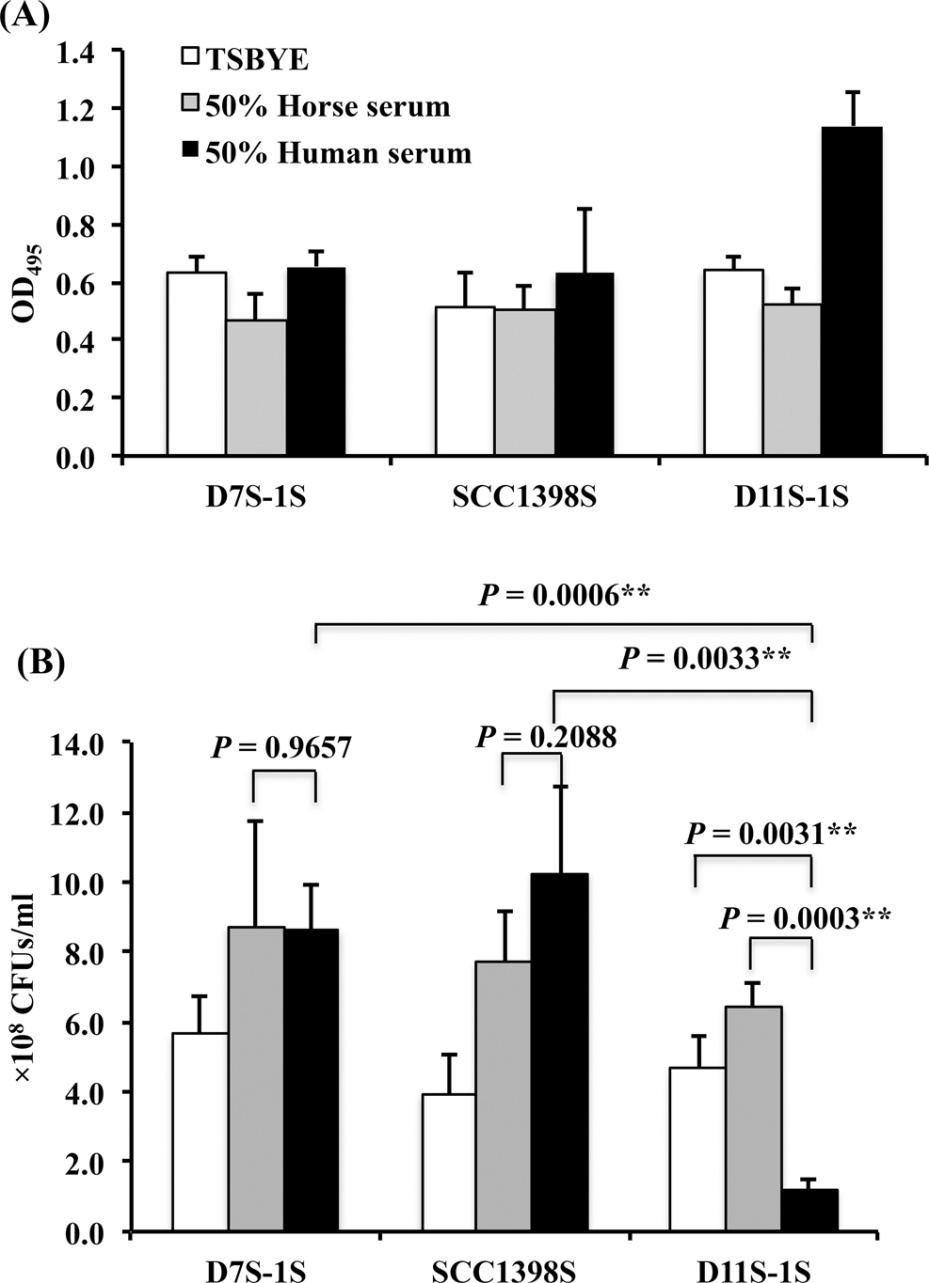
Comparison of final OD readings and colony forming units (CFUs) among three strains. D7S-1S, SCC1398S, and D11S-1S were grown in TSBYE, TSBYE + 50% human serum, or TSBYE + 50% horse serum for 23 h. All samples were prepared with a starting cell number of 0.5 × 1.0^8^–1.0 × 1.0^8^/ml. **(A).** D11S-1S reached the highest OD when incubated in human serum. The OD reading of D11S-1S in human serum was 2-fold higher than the readings for two low-responder strains incubated under the same condition. **(B).** D11S-1S incubated in 50% human serum for 23 h yielded the lowest number of recovered CFUs, representing only 18%-25% of the same strain incubated in either horse serum or TSBYE alone (*P* < 0.01). The recovered CFUs from D11S-1S grown in 50% human serum were less than 15% of either D7S-1S or SCC1398S incubated under the same condition (*P* < 0.01).

A parallel study comparing three strains based on both the final OD readings and the number of recovered CFUs was performed. The final OD of D11S-1S incubated in human serum for 23 h reached a reading twice that of the same strain grown in either TSBYE alone or in horse serum, and twice the final OD readings for two low-responder strains (D7S-1S and SCC1398S) grown under the same condition (Fig 3A). In contrast to the results from OD readings, the D11S-1S strain showed the lowest recovered CFUs after 23 h of incubation in human serum, yielding only 18% - 25% of the recovered CFUs for the same strain incubated in either horse serum or TSBYE alone (*P* < 0.01, unpaired *t*-test). The recovered CFU number for D11S-1S in human serum was only 15% of the recovered CFUs for either D7S-1S or SCC1398S incubated under the same condition (*P* < 0.01, unpaired *t*-test) (Fig 3B). There was no difference in recovered CFUs, when either D7S-1S or SCC1398S was exposed to human serum *versus* horse serum (*P* ≥ 0.2088, unpaired *t*-test).

**Fig 4:**
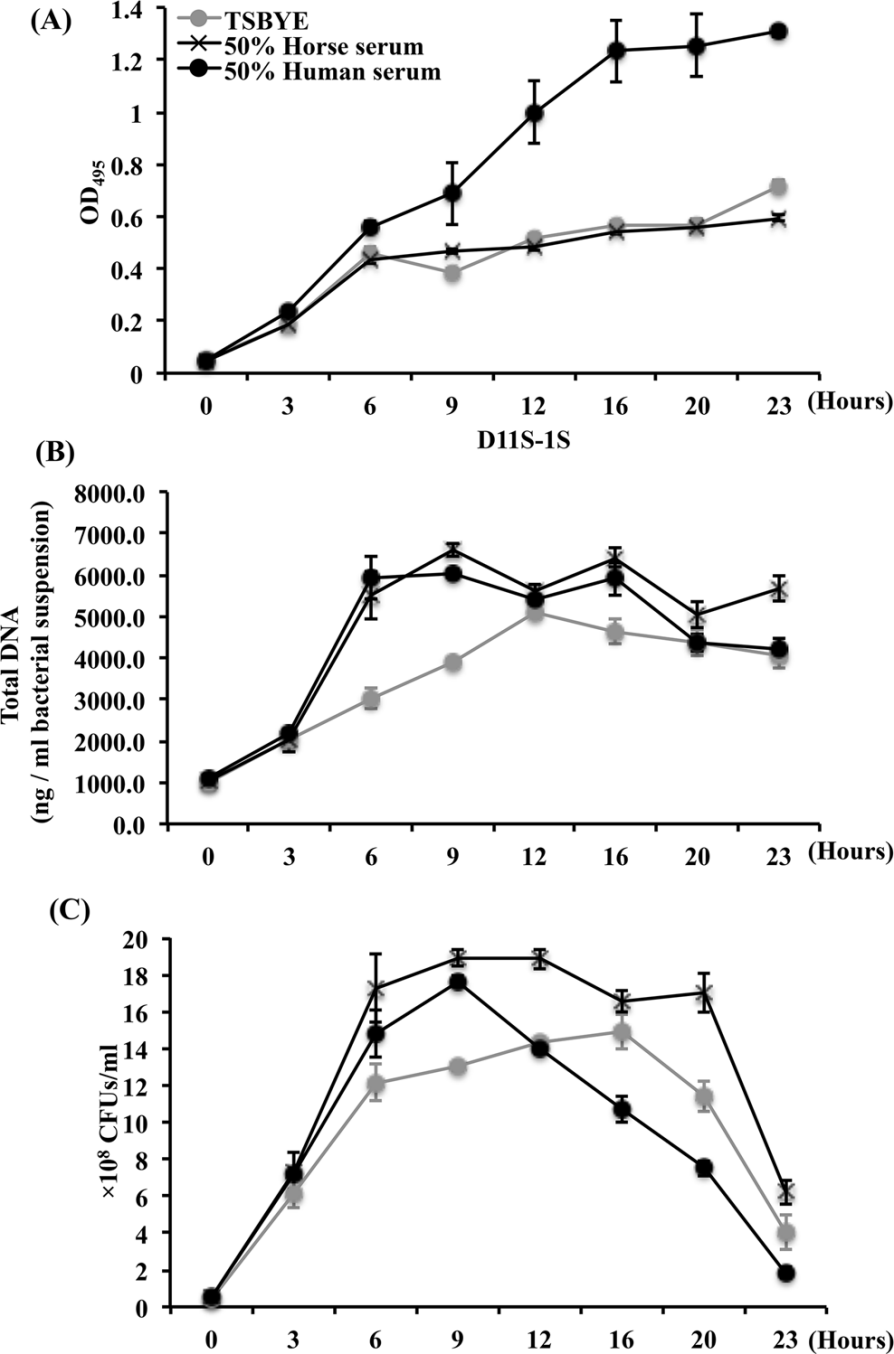
Comparison of growth curves of D11S-1S grown under three conditions. The incubation was performed at 37°C, with 5% humidified CO_2_. **(A).** D11S-1S demonstrated a second increase of OD readings after 9 h incubation in 50% human serum. **(B).** Both human serum and horse serum boosted the growth of D11S-1S within the early 6 h incubation, especially between 3rd and 6th hours based on the extracted total DNA. However, the growth curve based on extracted total DNA also indicated D11S-1S grown in 50% human serum reached the stationary phase after 6 h. **(C).** In contrast to a second increase of OD readings 9 h after exposure to human serum, the recovered CFU number decreased rapidly after 9-h incubation, indicating bacterial deterioration.

In addition, a 23-h continuously growth curves were obtained for D11S-1S at 3- to 4-h intervals based on OD, extracted total DNA, and recovered CFUs (Fig 4). A second rapid increase of turbidity occurred 9 h after exposure to human serum as expected (Fig 4A). However, the growth curve based on total DNA showed that serum boosted DNA replication only occurred during the first 6 h of incubation (Fig 4B), which included the replication of plasmids and phages. In addition, the growth curve based on recovered CFUs demonstrated a rapid decrease of viable cells 9 h after exposure to human serum (Fig 4C). Together, the data confirmed that the second increase in turbidity, occurred 9 h after D11S-1 exposure to human serum, was the result of cell deterioration and protein precipitation rather than cell growth.

### Transcriptomics identified accessory genes and core genes responding to human serum

Over 2000 genes with transcripts were identified respectively in three strains (D11S-1, SCC1398 and D7S-1) grown under three conditions (in TSBYE with human serum, TSBYE with horse serum or TSBYE alone). Up to 20% of them were accessory genes. Among the top 20 genes up-regulated the most by human serum in D11S-1, five of them were core genes that are present in all strains. Four of five top-responder core genes are within a four-gene operon art*PIQM*, showing 6–7 fold up-regulated by human serum in D11S-1 (Table 3). The ArtPIQM is a putative binding-protein transport system specific for arginine-uptake. The *artPIQM* operon was not up-regulated in SCC1398 and D7S-1 by human serum (S1 & S2 Tables). Fifteen of 20 were accessory genes with uncertain functions, including three putative Mu-like phage proteins; and these 15 accessory genes were up-regulated 4.8- to 6.6-fold by human serum, and 3.5- to 7.5-fold by horse serum, compared to TSBYE alone (Table 3). These 15 accessory genes are not present in the genome of D7S-1 and SCC1398. In contrast to 15 of 20 most up-regulated genes in D11S-1 by serum were accessory genes; only one accessory gene was found in the top 20 most up-regulated genes in D7S-1 (S1 Table), and none in SCC1398 (S2 Table).

**Table 3:** Top 20 most up-regulated genes by human serum in the high-responder strain D11S-1.

Genes*	Accessory	P-cluster***	Ratio	
	Genes**		Horse serum/TSBYE	Human serum/TSBYE
Arginine-binding periplasmic protein 1 (***artI***)	-	00709	**3.2**	**7.0**
Arginine transporter permease subunit (***artM***)	-	00731	**3.9**	**6.9**
Arginine transporter permease subunit (***artQ***)	-	01364	**4.0**	**6.8**
Transposase	+	02169	7.5	6.6
Hypothetical protein	+	02399	5.5	6.6
Hypothetical protein	+	07067	5.1	6.4
Arginine transporter ATP-binding subunit (***artP***)	-	00689	**3.1**	**6.2**
Hypothetical protein	+	02676	4.1	6.1
Hypothetical protein	+	02408	3.7	5.7
Metal dependent phosphohydrolase	+	03800	4.2	5.5
Hypothetical protein	+	02424	3.5	5.4
Hypothetical protein	+	03676	4.0	5.2
Putative sulfate transport protein (*cysZ*)	+	35311	4.2	5.2
Hypothetical protein	+	02396	4.2	5.0
Hypothetical protein	+	02326	6.9	5.0
Hemolysin A	-	00011	7.7	4.9
Mu-like phage gp25	+	02352	4.7	4.9
Hypothetical protein	+	02380	3.7	4.9
Mu-like phage gp26	+	02365	5.3	4.8
Mu-like phage gp27	+	02281	4.8	4.8

*The genes listed in Table 3 met both criteria that follow: a. The transcriptional level in human serum ≥ the median value among 2127 expressed genes based on RNA sequencing. b. The transcriptional level in human serum *versus* TSBYE ≥ 1.50.

The genes in bold belong to a four-gene operon *artPIQM*, a putative binding-protein transport system specific for arginine-uptake.

**Accessory genes (15 of 20): 15 genes are not present in two low-responder strains, D7S-1 and SCC1398.

***Detailed information of P-cluster is available in genomic database of *A*. *actinomycetemcomitans*: http://expression.washington.edu/genetable/script/gene_table_viewer.

Table 3 lists 20 genes, which were most up-regulated by human serum, as well as by horse serum to some extent. Therefore, additional screening was performed in D11S-1, and Table 4 lists the top 20 genes, which were only up-regulated by human serum (ratio of transcripts in human serum *versus* TSBYE ≥ 1.5), but not by horse serum (ratio of transcripts in horse serum *versus* TSBYE < 1.5). Among genes that were only up-regulated by human serum, five genes including *rpoH*, *rpoE*, *rseB*, *rseC* and *degQ* stood out (Table 4). These genes belong to three putative operons: *rpoH*, *rpoE-rseABC*, and *degQ*; and are specialized in stress responses. In contrast to D11S-1, none of these stress responder genes were up-regulated in the low-responder strain SCC1398 (S3 Table).

**Table 4:** Top 20 genes most up-regulated in the high-responder strain D11S-1 by human serum, but not by horse serum.

Genes*	Accessory	P-cluster***	Ratio	
	Genes**		Horse serum/TSBYE	Human serum/TSBYE
Hypothetical protein	-	00712	0.4	2.2
Sel1 domain-containing protein	-	01340	0.6	2.1
Putative tail fiber assembly protein	+	02272	1.4	2.1
**RNA polymerase factor sigma-32 (*rpoH*)**	-	00579	**0.6**	**2.0**
Glucitol operon repressor	-	00619	1.1	2.0
*recX*	-	00945	1.3	1.9
Transferrin-binding protein 1	-	02252	0.4	1.8
*nrdB*	-	00340	1.2	1.8
Glycerol-3-phosphate dehydrogenase	-	00141	1.4	1.8
**Periplasmic negative regulator of Sigma E** (***rseB***)	-	00447	**1.2**	**1.8**
**RNA polymerase sigma factor (*rpoE*)**	-	00845	**0.7**	**1.8**
Glucosamine—fructose-6-phosphate aminotransferase	-	00097	1.4	1.8
Hypothetical protein	-	01272	0.5	1.8
**Protease** (***degQ***)	-	00215	**0.6**	**1.7**
Putative antirepressor protein	+	02202	1.2	1.7
***rseC* protein**	-	02439	**1.1**	**1.7**
*recA*	-	00389	1.1	1.6
Helix-turn-helix containing protein	-	01355	0.9	1.6
Colicin uptake protein *tolR*	-	00978	0.9	1.6
Cytochrome c peroxidase	-	00213	1.3	1.6

*The genes listed in Table 4 met all three criteria that follow: a. The transcriptional level in human serum ≥ the median level among 2127 expressed genes based on RNA sequencing; b. The transcriptional level in human serum *versus* in TSBYE ≥ 1.5; c. Down-regulated by horse serum, or up-regulated by horse serum *versus* TSBYE less than 50%.

The genes in bold are specialized in stress response: *rpoE*, *rseB*, and *rseC* are within a putative, four-gene operon *rpoE-rseABC*.

**Accessory genes (2 of 20): both accessory genes are not present in the low-responder strains, D7S-1 and SCC1398.

***Detailed information of P-cluster is available in genomic database of *A*. *actinomycetemcomitans*: http://expression.washington.edu/genetable/script/gene_table_viewer.

Different transcriptional profiles of the up-regulated genes were shown in the high-responder strain D11S-1 (Tables 3 and 4), *versus* two low-responder strains D7S-1 (S1 Table) and SCC1398 (S2 & S3 Tables). In addition, multiple stress responders (highlighted in bold) in Table 4 were found only associated with the D11S-1 grown in human serum. Therefore, we hypothesized that the unusual growth phenomenon of D11S-1 in human serum is a series of consequences of activation of the sigma factor RpoE, a putative stress responder and global regulator.

### Genetic approaches demonstrated responses of the 132-bp *rpoE* promoter sequence of *A*. *actinomycetemcomitans* to hyperosmotic stress and human serum in *E*. *coli*

**Fig 5:**
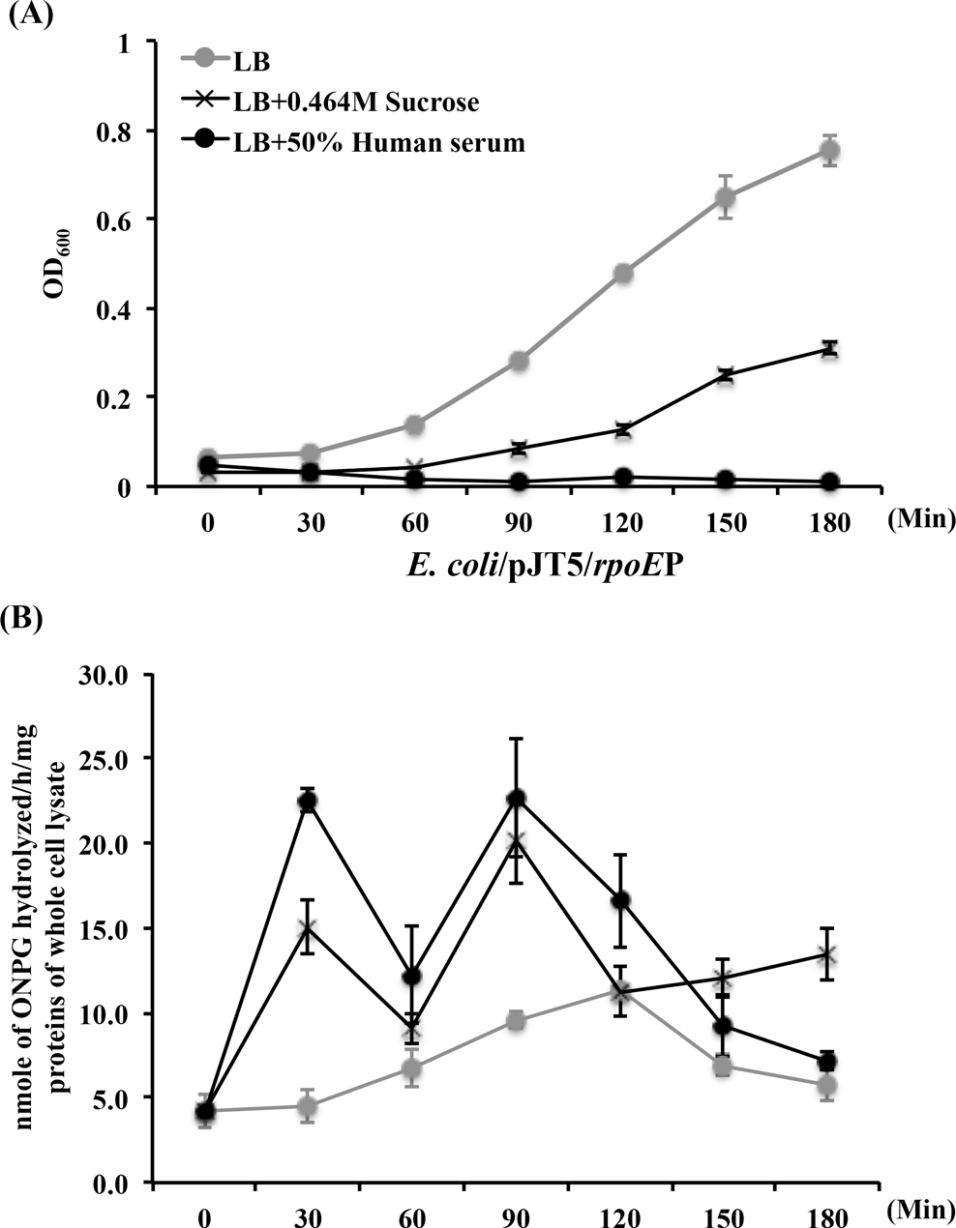
The *rpoE* promoter of *A*. *actinomycetemcomitans* responded to hyperosmotic stress and human serum in the host *E*. *coli*. The 132 bp putative *rpoE* promoter sequence was ligated into a promoter-less *lacZ* reporter construct pJT5 to generate a new construct pJT5/*rpoE*P. **(A).** Continuous OD readings of *E*. *coli*/pJT5/*rpoE*P grown under three conditions: standard Luria-Bertani lysogeny broth (LB); LB broth + 0.464M sucrose (to generate hyperosmotic membrane stress); and LB broth + 50% human serum. The presence of both 0.464M sucrose and human serum inhibited the growth of *E*. *coli*, especially the latter. **(B).** Continuous evaluation of β-galactosidase production was based on the amount of ONPG hydrolyzed per hour per mg of whole cell lysate proteins. The *rpoE* promoter sequence responded to both hyperosmotic stress and 50% human serum in a biphasic manner, with the initial elevated expression occurred within 30 min of incubation.

We initiated testing our hypothesis by cloning the putative 132 bp *rpoE* promoter sequence of D11S-1 (*rpoE*P) into a promoter-less *lacZ* reporter construct pJT5 (7,251 bp) to develop a new construct pJT5/*rpoE*P (7,383 bp) to examine how the *rpoE* gene responds to human serum. The plasmid pJT5/*rpoE*P responded positively to hyperosmotic stress (LB broth + 0.464 M sucrose) in *E*. *coli*, reaching the first peak of galactosidase production in 30 min (Fig 5B), accompanied with partial growth inhibition of the host of *E*. *coli* (Fig 5A). The same promoter also responded to 50% human serum, reaching the first peak of galactosidase production in 30 min (Fig 5B, accompanied with extensive growth inhibition of the host (Fig 5A). Interestingly, the expression of plasmid pJT5/*rpoE*P in *E*. *coli* was demonstrated in a biphasic manner (Fig 5B).

### Different responses of the 132-bp *rpoE* promoter sequence to human serum in a high- *versus* low-responder strain of *A*. *actinomycetemcomitans*

**Fig 6:**
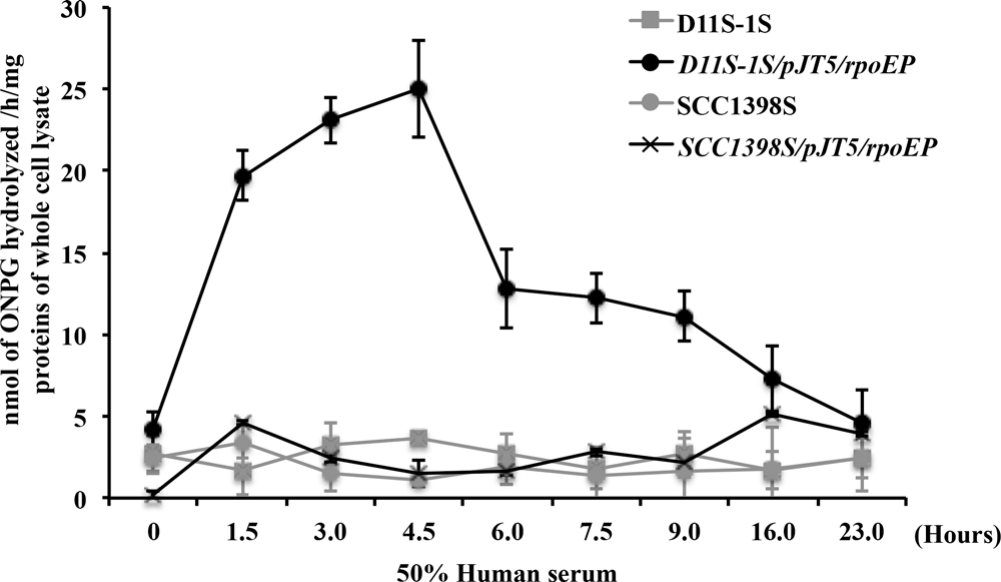
The *rpoE* promoter (*rpoE*P) of *A*. *actinomycetemcomitans* responded to human serum differently in the high-responder strain D11S-1S and the low-responder strain SCC1398S. The peak of β-galactosidase produced in D11S-1S/pJT5/*rpoE*P was around 5-fold higher than that in SCC1398S/pJT5/*rpoE*P. The ONPG production peaks also occurred at different time points: 4.5 h for D11S-1S/pJT5/*rpoE*P *versus* 16 h for SCC1398S/pJT5/*rpoE*P.

The 132 bp *rpoE* promoter sequence is conserved and 100% identical among most *A*. *actinomycetemcomitans* strains, including three representative strains. Therefore, the plasmid pJT5/*rpoE*P was only transformed into two *A*. *actinomycetemcomitans* strains, D11S-1S and SCC1398S, to test the new strains’ responses to human serum. Both strains demonstrated blue colonies in the presence of X-gal, indicating that the 132-bp promoter sequence successfully drove the expression of *lacZ*. Further comparison of the β-galactosidase production in D11S-1S/pJT5/*rpoE*P *versus* SCC1398S/pJT5/*rpoE*P demonstrated that the peak of β-galactosidase production in D11S-1S/pJT5/*rpoE*P was approximately 4-fold higher than in SCC1398S/pJT5/*rpoE*P (Fig 6). In addition, the peaks of galactosidase production occurred at different time points after exposure to human serum: 4.5 h for D11S-1S/pJT5/*rpoE*P, and 16 h for SCC1398S/pJT5/*rpoE*P (Fig 6). However, the transcriptional regulation of the four-gene operon *rpoE-rseABC* in the chromosome could be much more complex than what we observed using a simple *lacZ* reporter vector, because the replication of plasmids is independent of the chromosome.

### Transmission electron microscopy demonstrates differences in cellular morphology, vesicles secretion, and fimbria production

**Fig 7:**
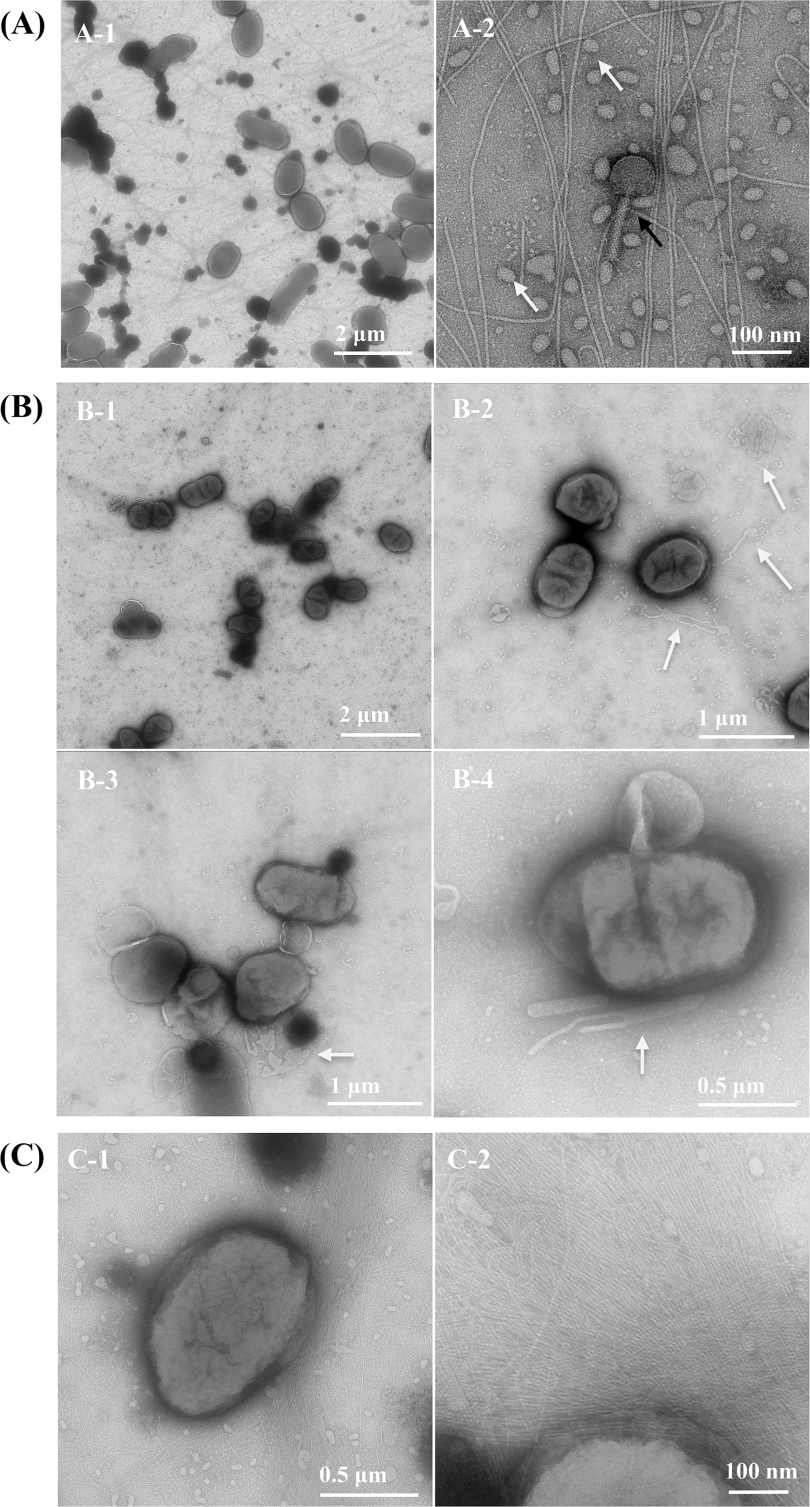
Transmission electron microscopy. The high-responder D11S-1 was grown on three different soft agars: TSBYE, TSBYE + 50% human serum, or TSBYE + 50% horse serum. **(A).** D11S-1 grown on TSBYE agar. Fimbriae, individual bacteriophage (↑) and vesicles (⇧) were clearly demonstrated (A-2). **(B).** D11S-1 grown on the agar with 50% human serum appeared to be smaller in size, with more convolutions on the surface, compared to the cells grown on TSBYE agar. Large numbers of outer membrane vesicles of multiple shapes and sizes in the intercellular space were also observed (indicated by ‘⇧’ in B-2, B-3, and B-4). **(C).** D11S-1 grown on 50% horse serum agar. Extraordinary numbers of fimbriae were observed associated with the cells grown under this condition.

Extra-cytoplasmic stress could lead to changes in cell morphology, vesicle secretion, and fimbria production. Transmission electron microscopy (TEM) of whole mount bacterial cell preparation allows the visualization and characterization of bacterial cell surface morphology while avoiding the fixation, dehydration, and embedding processes that can alter or damage cellular surface structures. Two- or three-adjacent colonies grown on soft agar were transferred directly onto the grid for observation, preserving cell morphology and surrounding structures. This TEM technique was used to observe D11S-1 cells grown under three conditions. Bacteriophages were clearly observed in the background (Fig 7A–2). Compared to cells grown on regular TSBYE agar (Fig 7A–1), D11S-1 cells grown on human serum agar appeared to be smaller in size with more convolutions on the surface and fewer fimbriae (Fig 7B). In addition, samples of D11S-1 grown in the presence of human serum revealed large numbers of outer membrane vesicles of multiple shapes and sizes in the intercellular space (Fig 7B). Compared to D11S-1 cells grown on either human serum or TSBYE agar, cells grown on horse serum agar appeared to synthesize an extraordinary number of fimbriae that were formed into bundles (Fig 7C).

### Confocal laser scanning microscopy demonstrates differences in cellular morphology and cell-cell interactions

**Fig 8:**
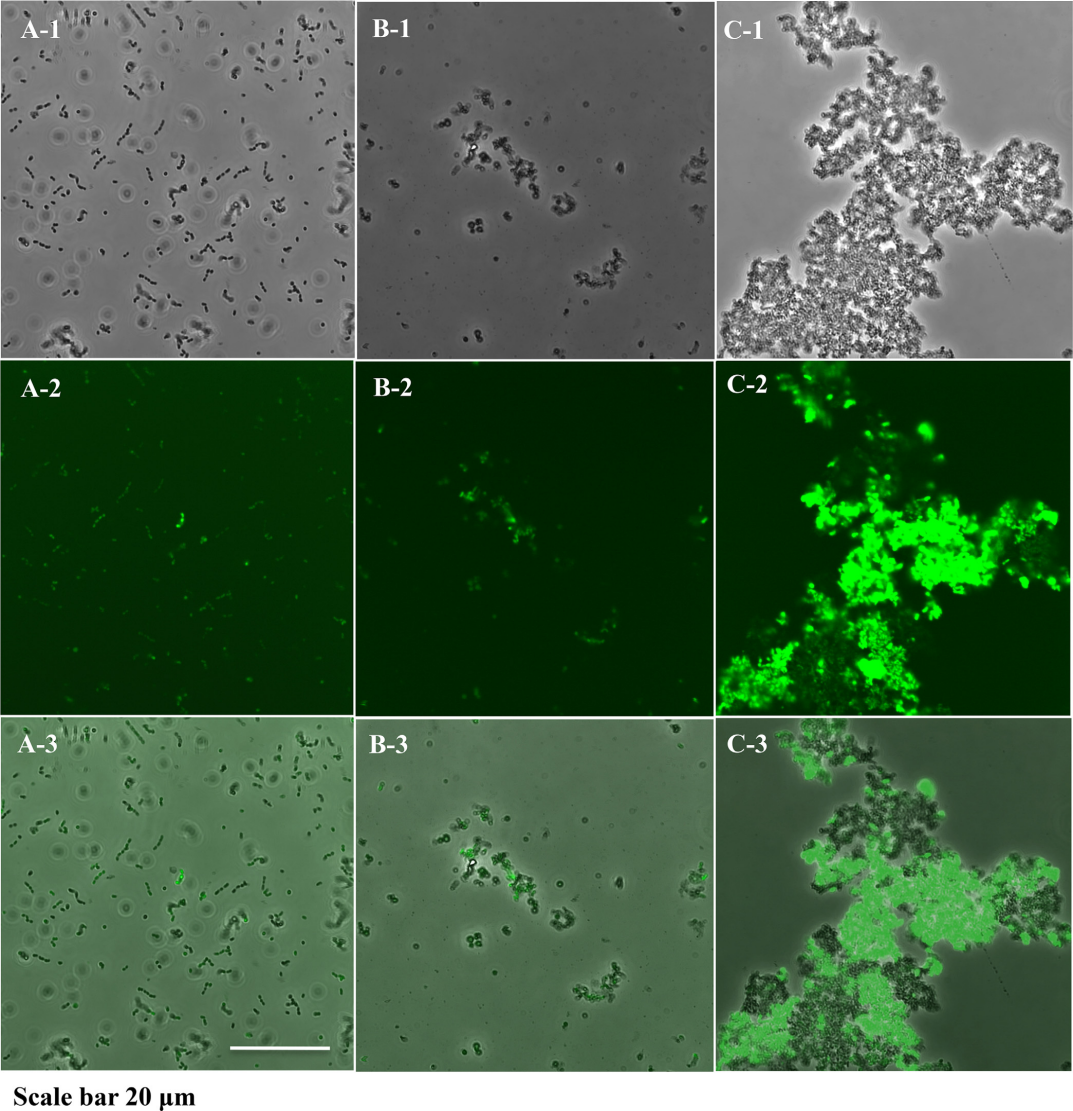
Confocal laser scanning microscopy. D11S-1S/pNP3 was grown under three conditions for 23 h. **(A).** D11S-1S/pNP3 grown in TSBYE broth. Bacteria appeared to be in typical coccobacillus shapes and clustered consistently in groups of 2–6 cells. A-1: phase contrast image; A-2: green fluorescent protein (GFP) filtered image; A-3: merged image of A-1 and A-2. The same description is applied to B and C. **(B).** D11S-1S/pNP3 grown in 50% human serum broth. Cells appeared to be in non-typical spherical shapes, and formed small aggregates randomly. **(C).** D11S-1S/pNP3 was grown in 50% horse serum broth. Bacteria formed large aggregates, and emitted stronger fluorescence compared to the cells grown in either TSBYE alone or 50% human serum broth.

D11S-1S/pNP3, a GFP-labeled D11S-1S strain, was used to demonstrate cell morphology and cell-cell interactions when bacteria were grown in liquid broths. D11S-1S/pNP3 grown in TSBYE broth tended to form clusters of 2–6 cells, where each cell displayed a typical coccobacillus shape (Fig 8A–1, 8A-2 and 8A-3). D11S-1S/pNP3 cells changed to a non-typical spherical shape with thick cellular boundaries and randomly formed small aggregates when they were grown in human serum broth (Fig 8B–1, 8B-2 and 8B-3). In contrast to the cells grown in either TSBYE or human serum broth, D11S-1S/pNP3 formed large aggregates and demonstrated high-intensity fluorescence when incubated in horse serum (Fig 8C–1, 8C-2 and 8C-3).

### SDS-PAGE identifies a novel band of ~25 kDa in the high-responder strain D11S-1

**Fig 9:**
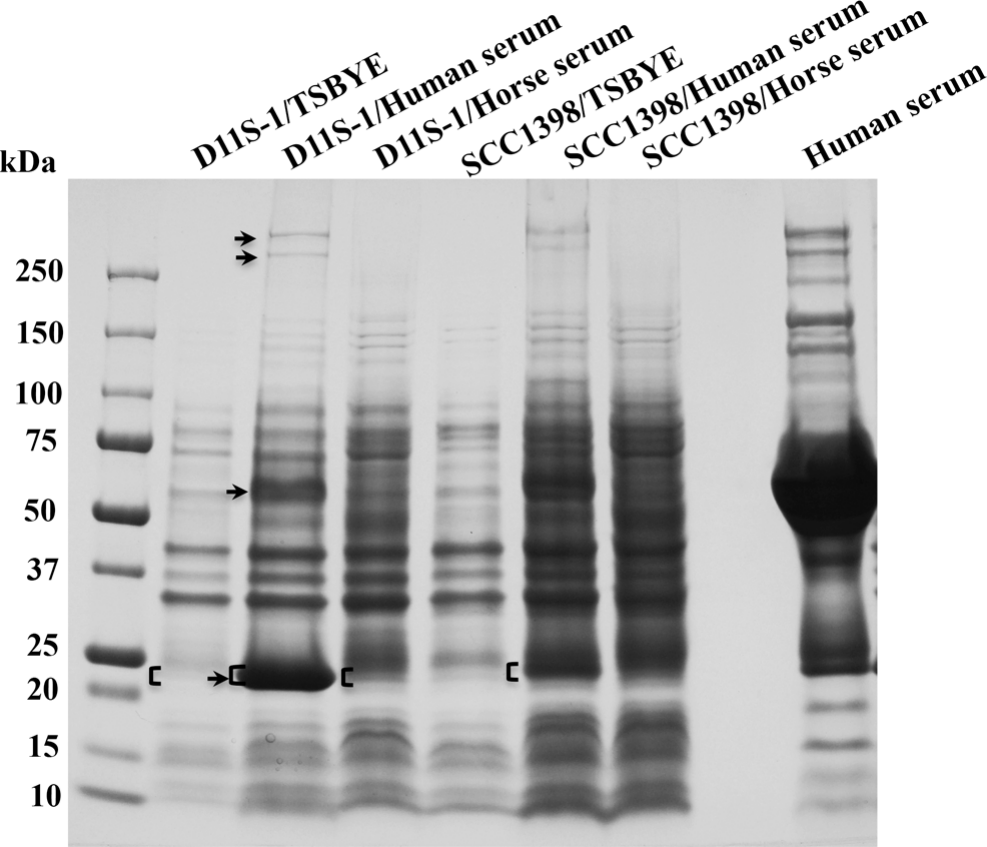
Colloidal blue stain of SDS-PAGE gel, and liquid chromatography/mass spectrometry (LC/MS) analysis. D11S-1 and SCC1398 were grown in TSBYE, TSBYE + 50% human serum, or TSBYE + 50% horse serum for 23 h using polystyrene tubes. Approximately the same numbers of cells were lysed, separated using a 4–15% gradient polyacrylamide gel, and stained. Four extra bands were associated with samples prepared using human serum: ~280 kDa, ~260 kDa, ~65 kDa and ~25 kDa (shown by ‘↑’). However, only the ~25 kDa band showed 3- to 10-fold stronger signal in the sample from D11S-1/human serum, compared to the samples from either SCC1398/human serum, D11S-1/horse serum or D11S-1/TSBYE (shown by square brackets ‘**[**‘). The ~25 kDa bands were excised and analyzed using a LC/MS system, and partial data were shown in Table 5.

A total of four extra bands were found associated with both D11S-1 and SCC1398 strains when grown in human serum. The corresponding proteins had approximate molecular masses of 280 kDa, 260 kDa, 65 kDa and 25 kDa. However, only the band that migrated with a molecular mass of ~25 kDa showed a 3- to 7-fold stronger signal in D11S-1/Human serum compared to SCC1398/Human serum, D11S-1/Horse serum, or D11S-1/TSBYE (Fig 9). Therefore, bands at the molecular mass of 25 kDa observed in D11S-1 and SCC1398S, each grown under three conditions, were excised for further liquid chromatography/mass spectrometry analysis.

### Liquid chromatography /mass spectrometry (LC/MS) analysis reveals phage replication

The top 10 identified proteins, based on the sequence coverage rates detected by LC/MS are listed in Table 5. The excised ~25 kDa band from the D11S-1/Human serum sample contained two proteins from human serum, ApoA1 (243 amino acids, ~28 kDa) and complement component C8 gamma chain (164 aa, ~18 kDa); two proteins from the bacteriophage S1249, D11S_2268 and D11S_2271; one protein from the plasmid S57 (Partition protein P-cluster 02649: 213 aa, ~23 kDa); and one accessory protein CysZ, P-cluster 35311, a putative sulfate transport protein (Table 5 and Fig 9). Both D11S_2268 (207 aa, ~24 kDa) and D11S_2271 (218 aa, ~25 kDa) are unique proteins associated with a bacteriophage that infected D11S-1. D7S-1 and SCC1398 were not found infected by phages. The accessory gene *cysZ* is also unique to D11S-1 among three representative strains. Consistently, the transcript of *cysZ* was 5-fold up-regulated in response to human serum (Table 3).

**Table 5:** Protein composition comparison of the ~25 kDa bands (Fig 9) from the high-responder strain D11S-1.

Proteins	P-cluster	Coverage (%)*		
		Human serum	Horse serum**	TSBYE
3-ketoacyl-(acyl-carrier-protein) reductase [D11S-1]	00698	90.1	90.5	92.2
Phosphate transport regulator [D11S-1]	01354	89.8	97.4	70.8
Apolipoprotein A1 [HUMAN]	N/A	81.7	N/A	N/A
Hypothetical protein D11S_2268 [Phage S1249]	15463	77.8	85.0	81.2
Putative solute/DNA competence effector [D11S-1]	00791	77.7	91.1	75.7
Partition protein [Plasmid S57]	02649	75.1	87.8	84.0
Triosephosphate isomerase TpiA [D11S-1]	00625	69.0	69.0	45.9
Putative sulfate transport protein CysZ [D11S-1]***	35311	67.2	67.6	Not detected
Hypothetical protein D11S_2271 [Phage S1249]	03578	65.6	78.4	Not detected
Complement component C8 gamma chain [HUMAN]	N/A	64.4	N/A	N/A
Hypothetical protein D11S_2218 [Phage S1249]	15413	Not detected	98.1	Not detected
Uracil phosphoribosyltransferase [D11S-1]	00789	56.7	90.9	71.6
Hypothetical protein D11S_2201 [D11S-1] ***	03676	Not detected	90.3	52.2
Cobyrinic acid a,c-diamide synthase [Plasmid S25]	02657	62.4	90.2	71.2
CmgB8 [D11S-1] ***	03526	54.0	80.4	49.4
Decarboxylase family protein Rossmann fold nucleotide-binding protein [D11S-1]	00851	Not detected	59.8	84.7
Acetyl-CoA carboxylase, biotin carboxyl carrier protein [D11S-1]	05408	Not detected	78.1	82.6
Ribonuclease T [D11S-1]	01369	Not detected	71.4	71.9

*The proteins are listed according to the coverage of the corresponding protein sequences detected by LC/MS analyses. The 10 proteins with the highest coverage in each of the three samples: D11S-1/Human serum (coverage: 90.1%-64.4%), D11S-1/Horse serum (Coverage: 98.1%-80.4%) and D11S-1/TSBYE (Coverage: 92.2%-70.8%), corresponding to bands labeled with square brackets ‘[‘ in Fig 9.

**The data generated from LC/MS did not match the horse serum database, and proteins from horse serum may be present.

***Accessory genes (3 of 18): *cysZ* (P-cluster35311) also showed 4- to 5-fold up-regulated transcripts in horse or human serum *versus* TSBYE (see Table 3).

N/A: not applicable.

Two of the proteins with the highest coverage in the sample of D11S-1/Human serum were not detected in D11S-1/TSBYE. They were CysZ and protein D11S_2271 located in the phage S1249. The sample D11S-1/Horse serum showed a protein profile similar to D11S-1/Human serum, except for the bound proteins from human serum (Table 5). Interestingly, three proteins detected in the ~25 kDa band from D11S-1/TSBYE, including P-cluster 00851 (189 aa, ~21 kDa); P-cluster 05408 (155 aa, ~16 kDa); and P-cluster 01369 (217 aa, ~24 kDa) were not detected in the sample D11S-1/Human serum, but were detected in the sample D11S-1/Horse serum (Table 5 and Fig 9).

## Discussion

*A*. *actinomycetemcomitans* is a fastidious oropharyngeal bacterium isolated from humans and rhesus monkeys, and host saliva alone cannot provide enough nutrients for its growth (Unpublished data). Serum, however, transports nutrients for both the host and colonizing microorganisms. During the disease state, gingival crevicular fluid (GCF) in the periodontal pocket contains mainly inflammatory exudates from serum. Either inflammatory exudates or the blood itself provides nutrients that are very different from TSBYE, the culture medium normally used to grow *A*. *actinomycetemcomitans in vitro*. Our work presents the first study designed to investigate the growth fitness, charactersitics and transcriptomic profiles of *A*. *actinomycetemcomitans* in human serum. The results may eventually lead to identification of unknown in vivo-specific virulence factors.

The most active genes that responded to human serum in the strain of D11S-1 were located in a putative *artPIQM* operon (Table 3). The *artPIQM* operon encodes a putative binding-protein-dependent transport system specific for L-arginine in *E*. *coli* [29]. The *artPIQM* is a core-gene operon with very similar promoter sequences among *A*. *actinomycetemcomitans* strains, suggesting that different up-stream regulatory components are involved. In contrast to only 25% of the 20 most active responder genes in D11S-1 were core genes (Table 3), more than 95% were core genes in 20 most active responder genes in D7S-1 and SCC1398 (data not shown), suggesting different pathways are activated in the high- and low-responder strains. Activation of the *artPIQM* operon in D11S-1 could be also one of the downstream responses following activation of the global regulator *rpoE* in this particular strain.

The *rpoE* gene is located in a four-gene operon *rpoE-rseABC* in *A*. *actinomycetemcomitans*, and the downstream genes *rseA*, *rseB* and *rseC* potentially regulate the activity of *rpoE* [30]. RpoE is considered as a global regulator that modulates the transcription of large numbers of downstream regulons and this regulation directly determines cellular division and survival in certain stress conditions [24, 31, 32]. DegQ is a putative ATP-independent protease and chaperone, which controls the quality of synthesized proteins in the periplasm. Both DegQ and RpoH are considered downstream regulons of RpoE [30, 33]. The *rpoE* gene mainly responds to extra-cytoplasmic (or envelope) stress that will affect the integrity of the membrane [30, 34]. The stress conditions that can potentially activate the *rpoE* response include: osmotic pressure, high temperature, stationary phase and infection by phages [30]. RpoE is also considered a virulence factor. Dysfunction of the *rpoE* gene or its regulons attenuated virulence of bacteria in species such as *Vibrio cholerae* and *Samolnella enterica* serovar Typhimurium [30, 35, 36]. The *rpoE* gene was activated in the high-responder D11S-1 by human serum, but not in the low-responders SCC1398 and D7S-1 also implied that the membrane components might be different in these two phenotypes.

Genetic approaches lent additional support to the statement that the *rpoE* of *A*. *actinomycetemcomitans* sensed and responded to the stress generated directly or indirectly in the presence of human serum, although the trigger components may be different for *E*. *coli* and *A*. *actinomycetemcomitans*. The inhibition of *E*. *coli* growth occurred immediately after the cells were exposed to 50% human serum (Fig 5A). The biphasic expression of plasmid pJT5/*rpoE*P either in the hyperosmotic condition or at the presence of human serum was observed only in the *E*. *coli* background (Fig 5B). It is likely that *E*. *coli* suppresses the non-endogenous *rpoE* promoter to some extent. In contrast, the growth of D11S-1 was accelerated within the first 6 h (Fig 4B and 4C); while the activity of the *rpoE* promoter remained high within the first 6 h in human serum as well (Fig 6). The data indicated that activation of *rpoE* might be required for the growth of D11S-1 in human serum. In support of this, a recent study showed that ApoA1, a major component of high-density lipoprotein (HDL), inhibits growth of *E*. *coli* and *Klebsiella pneumonia* by binding to bacterial membrane components: LPS and anionic vesicles [37]. Whether ApoA1 participates in triggering bacterial membrane stress, and activating *rpoE* in *A*. *actinomycetemcomitans* is unknown, although we did observe ApoA1 binding to *A*. *actinomycetemcomitans* (Fig 9 and Table 5).

Different responses of the same promoter sequence indicate that upstream activation of *rpoE* and the downstream RpoE-dependent regulons are different in the high- and low-responders. Consistently, RNA-seq data indicated that 75% of the most active responding genes in D11S-1 were accessory genes, including putative Mu-like phage proteins located in the chromosome (Table 3). Those accessory genes potentially participate in the activation of *rpoE* or are part of the downstream RpoE-dependent regulons.

D11S-1 grown in the presence of human serum appeared to have more convolutions compared to the cells grown on TSBYE or horse serum agar. Surface convolution changes usually suggest that cell envelope components are different. It has been demonstrated that a cell envelope protein, the morphogenesis protein C (MorC), is required for the convoluted surface structure and leukotoxin secretion in *A*. *actinomycetemcomitans* [38]. Other studies have also demonstrated that bacteria change their surface convolutions, when in stress conditions, including exposure to antimicrobial peptides [39, 40]. Increased numbers of large OMVs in the sample of D11S-1 grown in the presence of human serum suggest that the bacteria were in a stress condition [41].

Bacteriophage infection causes bacterial membrane perturbation and activates phage shock proteins, Psp in *E*. *coli* or SpxB (a homologue of Psp) in *Lactococcus lactis* [42] [43]. Similar to RpoE, Psp and its homologue are global regulators targeting extra-cytoplasmic stress [30]. Activation of global regulators change bacterial physiology to sustain the overall phage-mediated stress response [43]. Similar to RpoE, Psp is required for pathogens to grow in certain unfavorable environments and express virulence *in vivo* [44]. It is likely that the activation of *rpoE* in D11S-1 is either a response to the infection by phage S1249, or a response to the phage activation into lytic cycle by human serum.

Proteomic analysis was initiated to identify potential bacterial proteins with enhanced expression stimulated by human serum, as well as potential human serum proteins that bind to *A*. *actinomycetemcomitans*. The proteomic analysis showed that 2 of 10 proteins with the highest detection coverage in the sample from D11S-1/Human serum were associated with the 43 kb phage S1249. This observation together with the dramatic increase in total DNA for D11S-1 grown in human serum from hours 3 to 6 (Fig 4B), which was not reflected by the recovered CFUs (Fig 4C) suggest that phage replication is enhanced in the presence of serum.

Human serum-specific proteins, e.g. ApoA1 and complement components, differentiate human serum from horse serum. The serum used in this study was heat-inactivated. However, the presence of complement components in the ~25 kDa band of the sample from D11S-1/Human serum, and their absence from the sample of SCC1398/Human serum (data not shown) suggest that heat-inactivated complement proteins may bind to certain bacterial surface molecules. Whether complement components accelerate the interaction between phages or bacterial surface molecules, and human serum proteins requires further investigation.

Proteomic analysis also showed that an accessory protein CysZ (P-cluster35311), a putative sulfate transport protein was only expressed in the presence of serum, which was consistent with the transcriptomic analysis. Sulfur is an essential element in bacteria, and sulfate is the preferred source. However, sulfate uptake and metabolism is poorly understood in *A*. *actinomycetemcomitans*. We also observed ~5-fold up-regulated transcript encoding the putative Mu-like phage proteins (P-cluster 02352, P-cluster 02365 and P-cluster 02281), all of which are accessory genes located in the bacterial chromosome (Table 3). These accessory genes may participate in the replication of phage S1249. The strong signal of the ~25 kDa band from the sample of D11S-1/Human serum could be caused by over-expression of endogenous proteins of D11S-1, presence of the phage S1249, and/or pre-binding of human serum proteins that occurred during incubation (Fig 9). The absence of certain proteins expressed in the presence of human serum *versus* TSBYE suggests that different metabolism machineries are utilized by *A*. *actinomycetemcomitans*, when nutrients are different (Table 5).

## Conclusions

In summary, certain *A*. *actinomycetemcomitans* strains responded specifically to human serum by showing a second rapid increase of turbidity in the serum culture broth, which appears to be a consequence of cell deterioration and formation of protein aggregates. This phenomenon is likely triggered by the interaction between human serum and the bacterial membrane and/or structures associated with the bacterial membrane, e.g. phages. We hypothesize that this interaction subsequently activates an extra-cytoplasmic stress response that is controlled by *rpoE*. Activation of different accessory and core genes in the high- and low-responder strains appears to be subsequent events that follow two different pathways: with or without activation of the global regulator *rpoE*. The data suggest differences in the *in vivo* adaptations and pathogenesis among different strains of *A*. *actinomycetemcomitans*.

## Supporting Information

S1 Table Top 20 most up-regulated genes by human serum in the low-responder serotype a strain D7S-1. (DOCX)

S2 Table Top 20 most up-regulated genes by human serum in the low-responder serotype b strain SCC1398. (DOCX)

S3 Table Top 20 most up-regulated genes in the low-responder strain SCC1398 by human serum, but not by horse serum. (DOCX)
