# Supplementary material for: Human Serum-Specific Activation of Alternative Sigma Factors, the Stress Responders in Aggregatibacter actinomycetemcomitans
Source: PLoS One. 2016 Aug 4;11(8):e0160018. doi: 10.1371/journal.pone.0160018 (PMC4973924; doi:10.1371/journal.pone.0160018)
Supplement: S1 Table — (DOCX) [file pone.0160018.s001.docx]

**Supporting information**

**S1 Table.** Top 20 most up-regulated genes by human serum in the low-responder serotype a strain D7S-1.

| **Genes** | **Accessary** | **P-cluster** | **Ratio** | |
| --- | --- | --- | --- | --- |
|  | **Genes** |  | **Horse serum/TSBYE** | **Human serum/TSBYE** |
| Glycerol-3-phosphate dehydrogenase | - | 00141 | 5.3 | 3.7 |
| Hypothetical protein | - | 00887 | 0.7 | 2.9 |
| Membrane protein | - | 00712 | 2.1 | 2.7 |
| 5'/3'-nucleotidase SurE | - | 00686 | 2.5 | 2.4 |
| Sel1 domain-containing protein repeat-containingprotein | - | 01340 | 1.7 | 2.4 |
| Tyrosine-specific transporter | - | 00303 | 2.3 | 2.2 |
| Acetyl-CoA carboxylase biotin carboxylase subunit | - | 00189 | 3.0 | 2.1 |
| Fructose-1,6-bisphosphatase | - | 01252 | 1.2 | 2.0 |
| D-fructose-6-phosphate amidotransferase | - | 00097 | 5.4 | 2.0 |
| Hypothetical protein | - | 00900 | 2.4 | 1.9 |
| tRNA pseudouridine synthase D | - | 00424 | 2.6 | 1.9 |
| UDP-N-acetylglucosamine 1-carboxyvinyltransferase | - | 00277 | 2.6 | 1.8 |
| Hypothetical protein | - | 00684 | 1.8 | 1.8 |
| Dihydrolipoamide acetyltransferase component of pyruvate dehydrogenase complex | - | 05699 | 2.3 | 1.8 |
| Phosphocarrier protein HPr | - | 01085 | 1.4 | 1.8 |
| Helix-turn-helix containing protein | - | 01355 | 2.3 | 1.8 |
| Hypothetical protein**^#^** | + | 04810 | 1.5 | 1.8 |
| NADH pyrophosphatase | - | 01636 | 1.7 | 1.8 |
| 50S ribosomal protein L11 methyltransferase | - | 00544 | 1.7 | 1.8 |
| Dihydrolipoyl dehydrogenase | - | 09415 | 1.5 | 1.7 |

The genes met two criteria below:

a. the transcriptional levels in human serum ≥ the median value among 2,255 expressed genes based on RNA sequencing;

b. up-regulated by human serum *versus* TSBYE ≥ 50%.

All except one were core genes.
