# Supplementary material for: Human Serum-Specific Activation of Alternative Sigma Factors, the Stress Responders in Aggregatibacter actinomycetemcomitans
Source: PLoS One. 2016 Aug 4;11(8):e0160018. doi: 10.1371/journal.pone.0160018 (PMC4973924; doi:10.1371/journal.pone.0160018)
Supplement: S2 Table — (DOCX) [file pone.0160018.s002.docx]

**Supporting information**

**S2 Table.** Top 20 most up-regulated genes by human serum in the low-responder serotype b strain SCC1398.

| **Genes** | **Accessary** | **P-cluster** | **Ratio** | |
| --- | --- | --- | --- | --- |
|  | **Genes** |  | **Horse serum/TSBYE** | **Human serum/TSBYE** |
| Ferric iron binding protein | - | 00438 | 3.6 | 4.3 |
| Glucitol operon repressor | - | 00619 | 2.6 | 3.1 |
| 1,4-alpha-glucan branching enzyme | - | 00055 | 0.7 | 2.4 |
| Translation initiation factor IF-1 | - | 01103 | 1.8 | 2.2 |
| Periplasmic protein-probably involved in high-affinity Fe^2+^ transport | - | 00887 | 1.2 | 2.1 |
| Lipoprotein, putative | - | 01854 | 0.9 | 2.0 |
| Hypothetical protein | - | 03666 | 1.5 | 2.0 |
| D-fructose-6-phosphate amidotransferase | - | 00097 | 2.1 | 1.9 |
| 30S ribosomal protein S20 | - | 01076 | 1.5 | 1.9 |
| Universal stress protein (*uspE*) | - | 01305 | 1.7 | 1.8 |
| *dsrE* | - | 01431 | 2.3 | 1.7 |
| Hypothetical protein | - | 01033 | 1.0 | 1.7 |
| Leukotoxin | - | 00011 | 0.8 | 1.7 |
| *acrR* | - | 03109 | 2.5 | 1.7 |
| Cytochrome c-type protein (*torC*) | - | 00778 | 0.4 | 1.7 |
| 4-alpha-glucanotransferase | - | 00850 | 0.3 | 1.7 |
| PeriplasmiC nitrate reductase, diheme cytochrome c subunit | - | 00946 | 0.3 | 1.7 |
| Inner membrane protein | - | 00816 | 1.0 | 1.7 |
| Lipoprotein, putative | - | 06315 | 1.0 | 1.6 |
| Lipoprotein (*hlpB*) | - | 00757 | 1.9 | 1.6 |

The genes met two criteria below:

a. the transcriptional levels in human serum ≥ the median value among 1920 expressed genes based on RNA sequencing;

b. up-regulated by human serum *versus* TSBYE ≥ 50% .

All genes listed were core genes.
