# Supplementary material for: Human Serum-Specific Activation of Alternative Sigma Factors, the Stress Responders in Aggregatibacter actinomycetemcomitans
Source: PLoS One. 2016 Aug 4;11(8):e0160018. doi: 10.1371/journal.pone.0160018 (PMC4973924; doi:10.1371/journal.pone.0160018)
Supplement: S3 Table — (DOCX) [file pone.0160018.s003.docx]

**Supporting information**

**S3 Table.** Top 20 genes most up-regulated in the low-responder strain SCC1398 by human serum, but not by horse serum.

| **Genes** | **Accessary** | **P-cluster** | **Ratio** | |
| --- | --- | --- | --- | --- |
|  | **Genes** |  | **Horse serum/TSBYE** | **Human serum/TSBYE** |
| 1,4-alpha-glucan branching enzyme | - | 00055 | 0.7 | 2.4 |
| **Periplasmic protein-probably involved in high-affinity Fe^2+^ transport** | - | 00887 | 1.2 | 2.1 |
| Lipoprotein, putative | - | 01854 | 0.9 | 2.0 |
| Hypothetical protein | - | 01033 | 1.0 | 1.7 |
| Leukotoxin | - | 00011 | 0.8 | 1.7 |
| Cytochrome c-type protein (*torC*) | - | 00778 | 0.4 | 1.7 |
| 4-alpha-glucanotransferase | - | 00850 | 0.3 | 1.7 |
| PeriplasmiC nitrate reductase, diheme cytochrome c subunit | - | 00946 | 0.3 | 1.7 |
| Inner membrane protein | - | 00816 | 1.0 | 1.7 |
| Lipoprotein, putative | - | 06315 | 1.0 | 1.6 |
| **Iron ABC superfamily ATP binding cassette transporter, binding protein** | - | 00546 | 1.2 | 1.6 |
| Thiol-disulfide interchange protein | - | 00866 | 0.6 | 1.6 |
| Oxidoreductase domain protein | - | 00025 | 0.9 | 1.6 |
| **Ferric uptake regulator Fur** | - | 03772 | 1.0 | 1.6 |
| 2,3-bisphosphoglycerate-dependent phosphoglycerate mutase | - | 01547 | 1.2 | 1.6 |
| Cytochrome c nitrite reductase | - | 00155 | 0.3 | 1.5 |
| Hypothetical protein | + | 02384 | 0.4 | 1.5 |
| Short-chain dehydrogenase/reductase SDR | - | 01333 | 0.5 | 1.5 |
| **High-affinity Fe^2+^/Pb^2+^ permease** | - | 00082 | 0.6 | 1.5 |
| Fmn-dependent NADH-azoreductase | - | 03729 | 1.2 | 1.5 |

The genes listed in Table S-3 met three criteria below:

a. the transcriptional levels in human serum ≥ the median value among 1,920 expressed genes based on RNA sequencing;

b. up-regulated by human serum *versus* TSBYE ≥ 50%;

c. down-regulated by horse serum, or up-regulated by horse serum *versus* TSBYE less than 50%.

All except one were core genes.
